# Important Factors in Diagnosis and Treatment of Surgical Tuberculosis1Read at the forty-second annual meeting of the Medical Association of Central New York, held at Auburn, October 19, 1909.

**Published:** 1910-09

**Authors:** Martin B. Tinker

**Affiliations:** Ithaca, N. Y.; Assistant Professor of Surgery, Cornell University Medical College, Ithaca, N. Y.


### Important Factors in Diagnosis and Treatment of Surgical Tuberculosis.[1]

By MARTIN B. TINKER, S.B., M.D.,
Ithaca, N. Y.
Assistant Professor of Surgery, Cornell University Medical College, Ithaca, N. Y.

"MORE men fail because they don't look than because they don't know." Jenner expressed this idea, I believe, perhaps not in those exact words, but the situation remains unchanged in the many years from Jenner's time to our own. Men still fail to make correct diagnoses because they do not ask their patients to remove enough clothing and then use their eyes, ears and fingers, and possibly a tape measure. It seems unnecessary to enlarge on the importance of such obviously fundamental means of diagnosis; however there are other helps to correct diagnosis which I venture the majority of practitioners have never used, but which are of sufficient value to deserve more frequent application.

A positive diagnosis of tuberculosis can be reached by demonstrating the tubercle bacilli microscopically; and by the development of characteristic lesions after inoculation of guinea pigs. All recent graduates of reputable colleges know these methods, but do not use them as frequently as they should. For those of us who are too busy to bother with such work, good laboratories are numerous enough so that we no longer need to fail in diagnosis for lack of the aid of laboratory methods.

*The Tuberculin Test,* in connection with characteristic clinical features, gives an almost positive diagnosis and will clear up many doubtful cases. It is safe and simple enough so that any practitioner can use it in suitable cases. Several years ago, I reported my experience with 500 injections of tuberculin in surgical patients under my care at the Johns Hopkins Hospital. Since that time I have used the test in many additional cases and have seen no occasion to change my views. The Calmette ophthalmic test and von Pirquet cutaneous reaction, somewhat simplify the use of tuberculin. I have used them with satisfactory results in a limited number of cases and until there is good reason to change, shall continue to do so. Some writers question the reliability of both these tests and the safety of the Calmette test. Possibly the bad results from the Calmette test may have come from the use of unsuitable samples of tuberculin or faulty technic. Of the safety and reliability of the tuberculin test by injection, I can speak from long personal experience and careful study of the reported results of others. In doubtful cases, I should still

---

1. Read at the forty-second annual meeting of the Medical Association of Central New York, held at Auburn, October 19, 1909.

prefer it. It has been found of the greatest aid in determining the diagnosis in a number of cases in which there was doubt whether we were dealing with hysterical, traumatic or rheumatoid disease of the joints or tuberculosis in an early stage.

Several patients were sent out of the hospital without surgical treatment because of the reliance placed on the negative diagnosis furnished by the tuberculin test. Such affections of the hip, knee, spine, and the like, are by no means uncommon. It would be a serious mistake to allow early cases of joint tuberculosis to go without appropriate treatment at the only time in the course of the disease when such treatment is likely to prove helpful, and in this class of cases the test seldom fails to give important information. In several cases of tuberculosis of the kidney the test has shown the disease to be present and the diagnosis has been confirmed at operation, in cases in which repeated examinations of the urine have failed to demonstrate tubercle bacilli. No case of tuberculous glands of any part of the body failed to give a reaction, and none of the nine cases of Hodgkin's disease or the numberous cases of cysts of the neck, etc., which have sometimes been mistaken for tuberculosis of the lymph glands, reacted. Only two cases of osteomyelitis out of twenty-five reacted and there seemed to be fair evidence that the disease of the bone was tuberculous in at least one of these. No case of actinomycosis reacted to small doses and in one case the dose was raised to forty minims before reaction was produced. No reaction followed injection in several cases of syphilis. In many other cases in which the diagnosis was doubtful and in which early diagnosis was of great importance the test proved helpful; but it seems unnecessary to multiply examples.

From personal experience and a study of the writings of others, it seems to me that the evidence at hand is entirely favorable to tuberculin, both as regards its harmlessness and its reliability as a diagnostic agent. It remains yet to be proved that tuberculin rouses latent tuberculosis into activity or disseminates the disease, even in the enormous doses in which it was formerly given as a curative agent. The cases supposed to be of this kind are too few in number and the possibility of spontaneous spread of the disease must be remembered. The early writers who questioned the reliability of the test based their conclusions invariably on small numbers of cases, their statements are indefinite and the methods of preparing and administering tuberculin and of charting temperatures were no doubt equally imperfect. Those recent writers who have had most experience in its use are unanimously agreed as to its harmlessness and its high diagnostic value. If properly used in suitable cases, I am sure its use will prove most helpful in the diagnosis of tuberculosis from other surgical affections.

As aids to more correct diagnosis and treatment, the every day clinical use of laboratory methods and the tuberculin test can scarcely be too strongly urged. It is far more useful to our patients to make an early diagnosis that will help them to live, than to leave it to the pathologist to make ever so interesting a report postmortem. A few examples selected from my records will possibly give you a clearer idea of the class of cases in which I have found laboratory aids and the tuberculin test of special value.

*Diagnosis of Empyema from Tuberculous Conditions.*—A student from the college of civil engineering was sent to me by his uncle, a physician, with a history of pain in his chest, loss of weight, night sweats and some fever. An area of dulness extended over the entire lower right lung. There was slight expectoration and little cough. Other physicians who had been called in consultation naturally suspected tuberculosis. The tuberculin test was negative. We aspirated but got only a few drops of fluid. This fluid contained cocci, but no tubercle bacilli. It seemed evident that he did not have tuberculosis, and in spite of the fact that we got a little fluid on aspiration, a rib was resected, the pleural cavity freely opened and over a quart of pus evacuated. It would have done much harm to resect a rib in case of a pleura thickened by tuberculosis or tuberculosis of the lung. It would have been a greater mistake not to drain a collection of pus in the pleura, yet this is a common error. Many cases of empyema with cough, fever, emaciation, absence of breath sounds on ausculation and dulness on percussion, are passed over as tuberculosis without resort to any special diagnostic methods.

In the past few years, three patients have come to me suffering with empyema of long standing, who at one time or another had been thought to be suffering from lung tuberculosis in an incurable form. If laboratory methods, demonstration of tubercle bacilli in the suptum (if there is any sputum), demonstration of tubercle bacilli in aspirated fluid, or development of characteristic lesions on inoculation of guinea pigs or the tuberculin test; if one or more of these methods give a negative diagnosis, in such a case empyma should be suspected. Very often an aspirating needle is plugged with thick fibrin and we fail to get pus, but in such cases it is often the more imperative to open the pleura freely. With negative laboratory tests, resection of a rib can do little harm and it will save many a patient from prolonged illness, a crippled lung and in many cases, even life.

*Goiter with Pleural Effusion.*—A case of great interest, too rare a condition I judge, often to cause confusion, came to my notice recently. A woman of middle age was emaciated and weak, and had the physical signs of a collection of fluid in the pleural cavity. The fluid aspirated on at least twenty-five different occasions was free from pyogenic bacteria. She did not react to repeated tuberculin tests. No lesions developed in a guinea pig after inoculation. She had the classical symptoms of exophthalmic goiter and the question arose, whether the thyroid toxemia might be the cause of her effusion. Had there been any evidence of tuberculosis, it would have been manifestly unwise to meddle with the thyroid. With no evidence of any other possible cause for the effusion, it seemed advisable to attack the thyroid. Removal of the most enlarged lobe of the thyroid was carried out in stages, from one to two weeks apart; ligation of the superior thyroid artery, first on one side and then after an interval of a week, the other side; exposure of the gland and removal of the large lobe with packing of the wound, followed by careful closure about a week later. In this way the patient was brought safely through, the accumulation of fluid in the pleura has entirely ceased, and she has slowly but surely improved in health, gained over forty pounds in weight and now considers herself practically well. Without the assurance that the woman was not suffering from advanced tuberculosis which we could only get from our laboratory aids, operation and cure would have been out of the question.

*Breast tuberculosis* is also uncommon, but the diagnosis may be troublesome. A patient came to me recently who had a rapidly growing lump in the breast with slight enlargement of the axillary glands. The growth did not give the impression of a cancer, yet without some definite information and with the glandular enlargement, I should have thought it advisable to do the radical operation. The disadvantage of complete breast excision with thorough dissection of the axilla are apparent as compared with the less extensive operation necessary for breast tuberculosis; yet in an actual case it is impossible to decide which should be undertaken without some positive method of diagnosis.

*Treatment of Tuberculosis.*—Turning to the question of treatment of surgical forms of tuberculosis, it is safe to say that no class of cases requires better judgment. As with many other classes of diseases, it is often more important to decide what not to do, or how much to do than actually to accomplish what is needed. At least a dozen cases have come to my notice recently in which operation has been advised, when, in my judgment, suitable apparatus, general hygienic and dietetic treatment would be better. Another dozen cases had been temporised with when timely proper operative measures would have saved time, valuable function or even life. It would be conceited, indeed, to attempt to lay down rules which would make it possible entirely to avoid such mistakes. The factors influencing our success are so numerous and so complex; individual patients differ so greatly in vitality, and reaction to any form of treatment that a certain number of mistakes are unavoidable, however a discussion of such condition is sure to be helpful.

Our tuberculous cases may be divided roughly into three main classes: those best treated by non-operative measures,—splints, apparatus, rest, fresh air, abundant diet, suitable tonics and attention to other general measures. A second class in which operation should be advised, but with the greatest conservatism, in order to save useful organs and important functions. A third class of cases, in which the most thorough, painstaking, radical surgery with complete excision of foci or disease, will give our patients the best results. Factors of great importance influencing our choice of treatment, whether operative or non-operative, and if operative whether conservative or radical, are the extent and location of the disease; the age and social condition of our patients. Taking up a few of the more important forms of surgical tuberculosis, my own experience leads to advise as a general rule, radical operative treatment of glandular tuberculosis; conservative operation for tuberculosis of the genitourinary tract; and usually non-operative measures in suitable cases of bone and joint tuberculosis.

*Lymph Gland Tuberculosis.*—In lymphatic gland tuberculosis excision is recommended by practically all progressive surgeons, but not as frequently urged by general practitioners as would be to the best interests of their patients. Early excision would prevent widespread dissemination of the disease and death in many cases. Several fatal cases of meningitis and general miliary tuberculosis have come to my notice from such neglected cases of surgical tuberculosis. Not alone is there risk of spread of the disease to other organs; in practically all cases where the enlargement is considerable and persistent, suppuration occurs, with prolonged discharge and ugly scars as the result. Contrasted with the disfiguring and dangerous results of the "delay linger and wait policy," clean surgery gives rapid and certain cure, invisible scar and safety from spread of the disease. In over fifty cases of complete excision of glands of the neck in the past ten years, I have had no deaths, no recurrences, no spread of disease.

*Joint Tuberculosis.*—As to the age factor in joint tuberculosis, children almost invariably get a reasonably good, movable joint if an early diagnosis is made and proper treatment instituted. It is highly important that limping, night cries and painful joints be not disregarded or attributed to growing pains, rheumatism or injury. Growing is never a painful process. A healthy child seldom cries out at night without cause. Tuberculosis of joints in children is quite as common as rheumatism. The tuberculin test will frequently prove a most valuable aid in diagnosis. With advancing age, our treatment must be more and more radical. Patients past thirty seldom, if ever, entirely recover from joint tuberculosis under non-operative treatment. Resection of joints, removing most of the disease, gives far better results in these cases.

In advanced age, our treatment should frequently be even more radical. As an example, a man of seventy-two recently consulted me with regard to an enlarged painful ankle joint. He had been unable to use that foot in walking for several months and a few weeks previously an incision had been made and pus evacuated by a physician. There was strong tuberculous history, a wife and two children having died from lung tuberculosis. The tuberculin test was positive and although the *x*-ray showed that the disease was not extensive, it seemed safer to do an amputation. The patient will be able to get about with an artificial foot in a few weeks, where with any conservative treatment, he would have been weakened by pain and unable to get about for a long time. Probably at his advanced age, he would have succumbed to the disease long before anything had been gained by prolonged conservative treatment.

*Operative Measures.*—Factors necessary to success in all operative forms of tuberculosis are careful arrest of hemorrhage; clean, painstaking dissection; avoidance of smearing diseased tissue or pus into the healthy parts of the wound, care not to excise the epiphyseal line in operating for bone tuberculosis in children, thus arresting growth of the long bones; drainage as seldom as possible; as few sutures and as little ligature material left in the wound as will accomplish the result. These factors are of great importance for the highest success in all operative surgery, but they are of special importance in operating for tuberculosis, for if our tissues are damaged by rough handling; if they are crushed or bruised; if a diseased material is smeared by use of the curet, the healthy tissues will be unable to resist the invading bacteria and instead of doing our patients good, we may often do them harm. Success or failure is more dependent on a thorough knowledge of anatomy and painstaking and careful dissection on the part of the surgeon than on the extent of the disease and condition of the patient.

*Fresh Air and Overfeeding.*—Whatever form of treatment is selected as best suited in any particular case of surgical tuberculosis, general treatment is of the utmost importance. Fresh air, over feeding and rest are factors often neglected, but in every respect as indispensable as with tuberculosis of the lung. The patients, whether operated upon or treated by means of apparatus, should live in the open air, night and day, summer and winter. Patients often dislike this and raise all possible objections. They fear that they will take cold. They inquire if a damp air is not harmful and unless carefully watched, these simple and important factors will be neglected. In certain cases, the only way that this treatment can be successfully carried out is to send the patient to a hospital. In the case of children, it is sometimes necessary to forbid the parents to see them, for they bring in candy which will upset their digestion and spoil their appetites for plain and wholesome food.

As an example of what can be accomplished by these general hygienic measures: a child of two and a half years had been suffering from tuberculosis of the glands of the neck. Several suppurating glands had been opened by a physician and tonic remedies prescribed. The peritoneum was affected and on the inside of the knee joint a small abscess was opened. The child was extremely anemic, fretful, had no appetite and it was evident that without something was done he would die. He was taken to the hospital, the parents were not allowed to visit him, he was kept at perfect rest by strapping to a Bradford gas pipe frame, given plenty of eggs and milk and kept in the open air on the porch night and day. Within a very short time he began to improve. Without further surgery, his tuberculosis lesions healed and he is now rosy and fat and no one would suspect that he had ever suffered from tuberculosis.

The medical profession is apt to overlook the importance of these measures. Physicians do not insist that their surgical tuberculous patients shall be kept in the open air as they would with lung tuberculosis. They are not specific enough in arranging their diet, and very often the important factor of rest is entirely overlooked. Any patient suffering from tuberculosis, whether of the lung or any other part of the body, needs all of his energy to combat the disease. Those in charge of tuberculosis sanitariums have put their patients at rest. If patients are allowed to walk about and follow their usual occupations, the chances of rapid and perfect cure are very much reduced. Many years ago, Hilton in his classic book, "Rest in Pain," emphasised the importance of rest in many surgical conditions and his teachings still deserve reading and heeding.

Time is an especially important factor in the non-operative cases. Tuberculosis is a disease slow in development, as a rule, and it is equally slowly eradicated. Months certainly, and often years are required for complete cure. Patience on the part of the medical attendant and insistence that the treatment be faithfully followed is indispensable for success. In the Orthopedic hospitals, the importance of long continued treatment is usually recognised. In the Children's Hospitals bad cases of tuberculosis of the hip joint are kept in extension for at least six months and often for a year or a year and a half. A plaster paris spica extending to the ankle is then applied and the patient allowed to go about on crutches for six months; then a hip splint is used for six months to a year. This prolonged treatment gives perfect cures. Fresh air, over feeding, rest and time for nature to repair, are factors of the greatest importance in all forms of tuberculosis. This is specially true of surgical tuberculosis. A forceful lecturer whom some of you may have listened to in your student days, used to say that every hunch-back was a monument to the ignorance of the medical profession. My own feeling now is that it is not so much a question of ignorance as of indolence in many cases. It is an unpleasant and oftentimes a difficult task to argue and insist and fight with people to get them to follow directions explicitly, but without we are willing to sacrifice our own comfort and inclination to that extent, we are not likely to be emphatic and persistent enough to see that these important factors are understood and carried out.

---

**Diagnosis of Suppurative Disease of the Nasal Accessory Sinuses**

Alfred Braun, New York, gives a sketch of the anatomy of the accessory nasal sinuses. The cases of sinus disease may be divided into closed and open, that is, those in which there is no pus seen in the nose, and those in which a purulent discharge is seen in the nasal cavity. Most of the closed cases are acute, and the absence of pus is due to the swelling of the soft parts closing the openings of the sinuses. The symptoms are headache, with feeling of obstruction to the nose, and fever. There may be edema of the skin over the frontal sinus, or protrusion of the eye-ball due to pressure of the pus. Intranasal inspection shows little; there are edema and swelling of the turbinated bones, and polypi may be present. Transillumination and the *x*-rays are aids to diagnosis. The only positive diagnostic criterion is washing pus out of the sinus through the normal or an artificial opening. The anterior end of the middle turbinated bone must generally be removed to give access to the openings of the sinuses. Chronic closed sinus disease is rare. Open cases may be acute or chronic. They generally begin as closed cases, and pus appears after shrinkage of the tissues. The principal symptom in chronic cases is the presence of a profuse, offensive discharge from the nose. In ethmoidal disease the pus may dry in the nose and cause a foul odor. Headache may be absent, but is usually a prominent symptom. . . . . Treatment involves drainage and washing out of the affected cavity.—*Medical Record,* July 16, 1910.

